# Facing to “Duplicate Submission” As a Scientific Misconduct

**Published:** 2014

**Authors:** Seyyed Taha Yahyavi

**Affiliations:** Psychiatrist, Psychiatry and Behavioral Sciences Research Center, Mazandaran University of Medical Sciences, Mazandaran, Iran

**Dear Editor-in-Chief**

Iranian Journal of Psychiatry and Behavioral Sciences (IJPBS) recently has faced to some cases of unethical duplicate submissions. These papers were published in another journal before IJPBS decelerate the reviewer’s decisions. This can be classified as self-plagiarism or even redundant publication (1). 

Almost all journals and publishing guidelines prohibited this act (-). However, some authors continue to do it. I think the most important reason for it may be the urge of authors for publishing their study. In the absence of proper low and enforcement, some authors do this without any fear or shame.

Duplicate submission is an unethical act because:

1. When authors submit a paper for a specific journal, they transfer the right of publishing to that journal. Hence, two journals may be claimant it. It seems like a shopkeeper sell something into two people at the same time!
2. In instruction for authors of almost all journals, including IJPBS, duplicate submission is prohibited. If authors accept this instruction and do something in opposite way, a kind of lying is happened.
3. When authors submit a paper in two different journals simultaneously, some reviewers spend their times in reviewing a paper that would never be published in their journal. Hence, wrongdoer authors waste the reviewers times (5).
4. It is possible that two or more journals unknowingly and unnecessarily publish the same article (2).

What editors can do against this phenomenon?

1. Editors should never allow the wrongdoers to submit their paper in their journal again.
2. Editors of both involved journals should declare the name and affiliation of wrongdoers to make them “known” to the other journals and editors. Declaration of scientific fraud has been done before (6).
3. Editors should inform the supervisor or the chief of the department of wrongdoer and ask them to do the appropriate action against this phenomenon. Journals should emphasis on that the repetition of this act will have negative effect on the reputation of the affiliated department.
4. Editor of the affected journal should consult with the editor of the other journal that involved determining the proper course of action. It can be the rejection the paper by both journals (5).

At last, I want to ask you and all other editors-in-chief of scientific journals to face duplicate submitters decisively.
